# Como Comparar os Resultados Clínicos entre a Crioablação e a Ablação por Radiofrequência em Pacientes com Taquicardia por Reentrada Nodal Atrioventricular?

**DOI:** 10.36660/abc.20250078

**Published:** 2025-05-29

**Authors:** Naoya Kataoka, Teruhiko Imamura

**Affiliations:** 1 University of Toyama Toyama Japão University of Toyama, Toyama – Japão

**Keywords:** Taquicardia, Criocirurgia, Ablação por Radiofrequência

**Ao Editor**

A ablação de taquicardia de reentrada nodal atrioventricular (TRNAV) usando crioablação surgiu como uma alternativa viável à ablação por radiofrequência (RF) devido ao seu risco reduzido de induzir bloqueio atrioventricular permanente. No entanto, essa técnica necessita de considerações processuais específicas para minimizar a recorrência de arritmias. Os autores conduziram uma análise comparativa dos resultados clínicos entre a crioablação e a ablação por RF convencional, demonstrando que a crioablação foi associada à redução do tempo de fluoroscopia e não foi inferior à ablação por RF em termos de eficácia e viabilidade processual para o tratamento de TRNAV.^1^ No entanto, várias preocupações críticas merecem discussão adicional.

Na comparação das características basais, os intervalos átrio-his e his-ventricular foram observados como sendo maiores na coorte de crioablação.^1^ Isso levanta a possibilidade de que pacientes com intervalos PR basais prolongados foram selecionados preferencialmente para crioablação a fim de mitigar o risco de distúrbios de condução atriovenricular pós-procedimento. Se esse viés de seleção influenciou os resultados do estudo, ele deve ser reconhecido e explorado na discussão.

O significado clínico do período refratário efetivo do nó atriovenricular (PRENAV) permanece ambíguo. Se o PRENAV reflete o período refratário da via rápida, então a crioablação parece exercer um impacto maior na condução da via rápida em comparação à ablação por RF. Por outro lado, se o PRENAV representa a via lenta, é provável que seja influenciado pela infusão de isoproterenol administrada durante o procedimento, o que pode alterar a interpretação dos resultados.

Ao contrário da ablação por RF, cujo local de tratamento é guiado por marcos anatômicos, a crioablação frequentemente apresenta desafios na localização precisa do local terapêutico ideal. Os autores empregaram sistemas de mapeamento tridimensional recentemente inovados para identificar as zonas de ablação alvo para a crioablação precisamente?^2 , 3^ Este ponto é particularmente significativo, dado que três pacientes apresentaram arritmias recorrentes após o procedimento.
