# Supplementary material for: Como Comparar os Resultados Clínicos entre a Crioablação e a Ablação por Radiofrequência em Pacientes com Taquicardia por Reentrada Nodal Atrioventricular?
Source: Arq Bras Cardiol. 2025 May 29;122(5):e20250078. [Article in Portuguese] doi: 10.36660/abc.20250078 (PMC12212367; doi:10.36660/abc.20250078)
Supplement: Translation

# Translation: How to Compare Clinical Outcomes between Cryoablation and Radiofrequency Ablation in Patients with Atrioventricular Nodal Reentrant Tachycardia?

**DOI:** 10.36660/abc.20250078i

**Authors:** Naoya Kataoka, Teruhiko Imamura

**Affiliations:** 1 University of Toyama Toyama Japan University of Toyama, Toyama – Japan

**Keywords:** Tachycardia, Cryosurgery, Radiofrequency Ablation

**To Editor**

The ablation of atrioventricular nodal reentrant tachycardia (AVNRT) using cryoablation has emerged as a viable alternative to radiofrequency (RF) ablation due to its reduced risk of inducing permanent atrioventricular block. However, this technique necessitates specific procedural considerations to minimize the recurrence of arrhythmias. The authors conducted a comparative analysis of clinical outcomes between cryoablation and conventional RF ablation, demonstrating that cryoablation was associated with reduced fluoroscopy time and was non-inferior to RF ablation in terms of efficacy and procedural feasibility for AVNRT management.^1^Nonetheless, several critical concerns merit further discussion.

In the comparison of baseline characteristics, atrium-his and his-ventricular intervals were observed to be longer in the cryoablation cohort.^1^This raises the possibility that patients with prolonged baseline PR intervals were preferentially selected for cryoablation in order to mitigate the risk of post-procedural atrioventricular conduction disorders. If this selection bias influenced the study's outcomes, it should be acknowledged and explored in the discussion.

The clinical significance of the atrioventricular node effective refractory period (AVNER) remains ambiguous. If AVNER reflects the refractory period of the fast pathway, then cryoablation appears to exert a greater impact on fast pathway conduction compared to RF ablation. Conversely, if AVNER represents the slow pathway, it is likely influenced by the isoproterenol infusion administered during the procedure, which could alter the interpretation of results.

Unlike RF ablation, whose treatment site is guided by anatomical landmarks, cryoablation often poses challenges in accurately localizing the optimal therapeutic site. Did the authors employ recently innovated three-dimensional mapping systems to identify the target ablation zones for cryoablation precisely?^2 , 3^This point is particularly significant given that three patients experienced recurrent arrhythmias following the procedure.
